# Editorial: Ethylene's Role in Plant Mineral Nutrition

**DOI:** 10.3389/fpls.2016.00911

**Published:** 2016-06-27

**Authors:** Francisco J. Romera, Aaron P. Smith, Rafael Pérez-Vicente

**Affiliations:** ^1^Department of Agronomy, Edificio Celestino Mutis (C-4), University of CórdobaCórdoba, Spain; ^2^Department of Biological Sciences, Louisiana State UniversityBaton Rouge, LA, USA; ^3^Department of Botany, Ecology and Plant Physiology, Edificio Celestino Mutis (C-4), University of CórdobaCórdoba, Spain

**Keywords:** ethylene, heavy metals, mineral nutrition, nodulation, nutrient deficiency responses, salinity

Ethylene is a gaseous plant hormone involved in many aspects of plant life, including seed germination, flower senescence, abscission, and fruit ripening (Abeles et al., 1992). It also plays a very important role in the responses of plants to both biotic and abiotic stresses (Abeles et al., 1992; Shakeel et al., 2013; Kazan, 2015). The production of ethylene is tightly regulated by internal signals, and usually increases in response to biotic (e.g., pathogen attack) and abiotic stresses, such as mechanical stress, hypoxia, chilling, and nutritional disorders (Abeles et al., 1992; Lynch and Brown, 1997; Concellon et al., 2005; Zheng et al., 2008; Geisler-Lee et al., 2010; Iqbal et al., 2013; García et al., 2015).

In processes related to mineral nutrition, ethylene has been implicated in the regulation of physiological and morphological responses to nutrient deficiencies; in nodulation of legume plants; in salt tolerance responses; and in responses to heavy metals (Abeles et al., 1992; Lynch and Brown, 1997; García et al., 2015).

This research topic updates recent results relating ethylene to different aspects of plant mineral nutrition. It includes 10 reviews and 2 original articles: 7 reviews are related to nutrient deficiencies (Khan et al.; Lucena et al.; Schachtman; Song and Liu; Wawrzynska et al.; González-Fontes et al.; Neumann), 1 to nodulation (Guinel), 1 to salt tolerance (Tao et al.), and 1 to heavy metals (Keunen et al.); 1 original article is related to Fe (iron) deficiency (Ye et al.), and the other one to N (nitrogen) deficiency (De Gernier et al.).

The role of ethylene in the regulation of responses to nutrient deficiencies was introduced in the nineties, when some studies showed an implication of ethylene in the regulation of physiological and/or morphological responses to Fe and P (phosphorus) deficiency (Romera and Alcántara, 1994; Lynch and Brown, 1997). In the last years, the role of ethylene has been extended to other nutrient deficiencies, such as K (potassium) deficiency, S (sulfur) deficiency, and others (Iqbal et al., 2013; García et al., 2015). The relationship between ethylene and other processes related to mineral nutrition (nodulation, salinity and heavy metals) has also been known for many years (Abeles et al., 1992; Lynch and Brown, 1997).

Since nutrient deficiencies cause stress to plants and stress promotes ethylene synthesis (Abeles et al., 1992), most plant species increase ethylene production under different deficiencies (Lucena et al.; Schachtman; Song and Liu; Wawrzynska et al.). This higher ethylene production is generally associated with increased transcript abundance for genes involved in ethylene biosynthesis and signaling (Lucena et al.; Schachtman; Song and Liu; Wawrzynska et al.; Neumann). Moreover, the mitogen-activated protein kinases 3 and 6 (MPK3/MPK6), that can regulate ethylene production, increase under Fe deficiency (Ye et al.) or under heavy metal stress (Keunen et al.). In the case of N, the relationship between its deficiency and ethylene production seems to be complex, affected by the degree of the deficiency and the plant genotype (Khan et al.; De Gernier et al.).

Similarly to nutrient deficiencies, both salinity stress and heavy metal stress also cause higher ethylene production and increased transcription of ethylene biosynthesis and signaling genes (Tao et al.; Keunen et al.). In the nodulation process, ethylene production increases early in the symbiosis (Guinel).

In general, ethylene plays positive roles in the activation of responses of plants to nutrient deficiencies (Khan et al.; Lucena et al.; Schachtman; Song and Liu; Wawrzynska et al.; Ye et al.; De Gernier et al.; González-Fontes et al.; Neumann), to salinity stress (Tao et al.), and to heavy metal stress (Keunen et al.), while it is considered a negative regulator of the nodulation process (Guinel). Despite these generally accepted roles, conflicting results have also been reported. As examples, some research has shown that ethylene insensitive plants are more tolerant to salinity (Tao et al.) or to heavy metal stress (Keunen et al.) than corresponding wild types. To explain these contradictory results, it should be taken into account the different experimental conditions used, including plant material, dosage, days of treatments, etc. (Keunen et al.). Additionally, it should be considered that excessive ethylene could inhibit plant growth and development (Tao et al.; Keunen et al.).

In relation to nutrient deficiencies, ethylene has been implicated in the activation of both physiological and morphological responses, such as enhanced ferric reductase activity under Fe deficiency (Lucena et al.), enhanced acid phosphatase activity under P deficiency (Song and Liu), upregulation of the HAK5 potassium transporter under K deficiency (Schachtman), development of root hairs under Fe, K, B (boron) or P deficiency (Lucena et al.; Schachtman; González-Fontes et al.; Neumann), and development of cluster roots under Fe or P deficiency (Lucena et al.; Neumann). In relation to salinity stress, ethylene has been implicated in the regulation of Na^+^/K^+^ homeostasis (Tao et al.); and in relation to heavy metal stress, in the network leading to glutathione and phytochelatin synthesis (Keunen et al.). In the nodulation process, ethylene has been implicated in most of the steps leading to a mature nodule and even in nodule senescence (Guinel).

The participation of ethylene in all the processes described above suggests it should act in conjunction with other signals, and/or perhaps through different transduction pathways, to confer specificity to the different responses. Both possibilities are reflected in the reviews included in this research topic. As examples, ethylene interacts with auxin and phloem signals to regulate Fe deficiency (Lucena et al.) and P deficiency responses (Song and Liu; Neumann); with ABA to regulate responses to salinity (Tao et al.); and with ROS to regulate responses to K deficiency (Schachtman) and heavy metals (Keunen et al.). On the other hand, Lucena et al. present evidence suggesting that ethylene regulates different responses to Fe deficiency through distinct transduction pathways, which is in agreement with recent proposals about ethylene signaling (Shakeel et al., 2013; Zhang et al.).

Despite the specificity conferred by different signals, the common participation of ethylene in different processes related to plant mineral nutrition could partly explain the frequent cross talks among nutrient deficiency responses (Lucena et al.) and between salinity and nutrient deficiencies (Tao et al.).

In conclusion, this research topic, by putting together different nutritional aspects affected by ethylene, tries to pave the way for future research about the role of this simple but fascinating hormone on plant mineral nutrition.

## Author contributions

For the Editorial, AS reviewed the works about P and K; RP reviewed the works about N and B; and FR reviewed the rest of works. A draft of the Editorial was first written by Dr. Romera and then Dr. Smith and Dr. Pérez-Vicente revised and modified it to get the final version.

### Conflict of interest statement

The authors declare that the research was conducted in the absence of any commercial or financial relationships that could be construed as a potential conflict of interest.

## References

[B1] Abeles F. B. Morgan P. W. Saltveit M. E. (1992). Ethylene in Plant Biology, 2nd Edn. San Diego, CA: Academic Press.

[B2] Concellon A. Anon M. C. Chaves A. R. (2005). Effect of chilling on ethylene production in eggplant fruit. Food Chem. 92, 63–69. 10.1016/j.foodchem.2004.04.048

[B3] García M. J. Romera F. J. Lucena C. Alcántara E. Pérez-Vicente R. (2015). Ethylene and the regulation of physiological and morphological responses to nutrient deficiencies. Plant Physiol. 169, 51–60. 10.1104/pp.15.00708 26175512 PMC4577413

[B4] Geisler-Lee J. Caldwell C. Gallie D. R. (2010). Expression of the ethylene biosynthetic machinery in maize roots is regulated in response to hypoxia. J. Exp. Bot. 61, 857–871. 10.1093/jxb/erp362 20008461 PMC2814119

[B5] Iqbal N. Trivellini A. Masood A. Ferrante A. Khan N. A. (2013). Current understanding on ethylene signaling in plants: the influence of nutrient availability. Plant Physiol. Biochem. 73, 128–138. 10.1016/j.plaphy.2013.09.011 24095919

[B6] Kazan K. (2015). Diverse roles of jasmonates and ethylene in abiotic stress tolerance. Trends Plant Sci. 20, 219–229. 10.1016/j.tplants.2015.02.001 25731753

[B7] Lynch J. P. Brown K. M. (1997). Ethylene and plant responses to nutritional stress. Physiol. Plant. 100, 613–619. 10.1111/j.1399-3054.1997.tb03067.x

[B8] Romera F. J. Alcántara E. (1994). Iron-deficiency stress responses in cucumber (*Cucumis sativus* L.) roots. A possible role for ethylene? Plant Physiol. 105, 1133–1138. 12232270 10.1104/pp.105.4.1133 PMC159441

[B9] Shakeel S. N. Wang X. Binder B. M. Schaller G. E. (2013). Mechanisms of signal transduction by ethylene: overlapping and non-overlapping signalling roles in a receptor family. AoB Plants 5:plt010. 10.1093/aobpla/plt010 23543258 PMC3611092

[B10] Zheng C. Wang W. Huang Z. Hara T. (2008). Mechanical stress modifies endogenous ethylene and gibberellin production in chrysanthemum. Acta Hort. 766, 103–107. 10.17660/ActaHortic.2008.766.11

